# Histological and ultrastructural characterization of the dorso-ventral skin of the juvenile and the adult starry puffer fish (*Arothron stellatus,* Anonymous 1798)

**DOI:** 10.1186/s12917-023-03784-0

**Published:** 2023-10-24

**Authors:** Fatma A. Madkour, Ahmed M. Abdellatif, Yassein A. Osman, Ramadan M. Kandyel

**Affiliations:** 1 Department of Anatomy and Embryology, Faculty of Veterinary Medicine, South Valley University, Qena, 83523 Egypt; 2 Department of Anatomy and Embryology, Faculty of Veterinary Medicine, Mansoura University, Mansoura, 35516 Egypt; 3 Department of Fisheries, Fish Population Dynamic Lab, National Institute of Oceanography and Fisheries, Hurghada, Red Sea Egypt; 4 Department of Zoology, Faculty of Science, Tanta University, Tanta, Egypt; 5 Department of Biology, Faculty of Arts and Sciences, Najran University, Najran, Saudi Arabia

**Keywords:** Club cells, Keratinocytes, Microridges, Mucous cells, Taste buds, Starry puffer fish, Ultrastructure

## Abstract

**Background:**

The starry puffer fish (*Arothron stellatus,* Anonymous, 1798*)* is a poisonous tetradontidae fish inhabiting the Red sea. The skin constitutes an important defense against any external effects. The study aims to characterize the dorso-ventral skin of the juvenile and the adult starry puffer fish using light and scanning electron microscopies. Twenty specimens of juvenile and adult fresh fishes were used.

**Results:**

The scanning electron microarchitecture of the skin of the juvenile and adult fish showed delicate irregular-shaped protrusions, and well-defined bricks-like elevations on the dorsal side and interrupted folds as well as irregular-shaped protrusions on the ventral side. In adult fish, the patterned microridges of the superficial and deep epithelial cells (keratinocytes) were larger and well-defined in the dorsal skin than in the ventral side, the contrary was seen in the juvenile fish. The microridges were arranged in a fingerprint or honeycomb patterns. The openings of the mucous cells were more numerous in the dorsal skin in both age stages but more noticeable in adult. Furthermore, the sensory cells were more dominant in the juveniles than the adults. The odontic spines were only seen in adult. Histologically, few taste buds were observed in the epidermis of the dorsal skin surface of the adult fish. Both mucous and club cells were embedded in the epidermis of the juvenile and adult fish with different shapes and sizes. Melanophores were observed at the dorsal skin of both juvenile and adult fishes while fewer numbers were noticed at the ventral surfaces. Several dermal bony plates with different shapes and sizes were demonstrated in the skin of both adult and juvenile fishes.

**Conclusion:**

The structural variations of skin of the juvenile and adult fishes may reflect the various environmental difficulties that they confront.

## Introduction

**Fig. 1:**
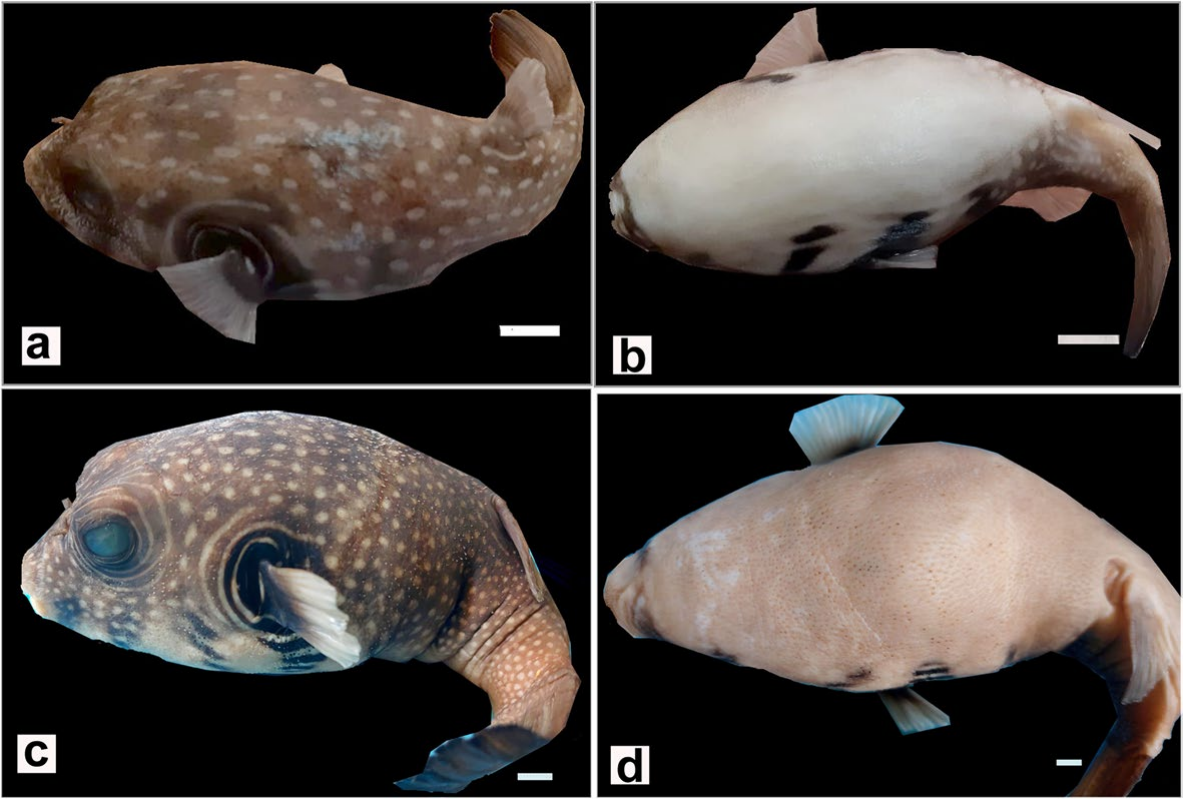
Photographs of juvenile (**a**, **b**) and adult (**c**, **d**) starry puffer fish showing the normal appearance of the skin of dorsal (**a**, **c**) and ventral (**b**, **d**) aspects of their bodies. Scale bar = 1 cm

Starry puffer fish (*Arothron stellatus,* Anonymous, 1798) is a member of the family tetradontidae [1]. It is a highly poisonous species found throughout the tropical and subtropical waters of the Indian Ocean, Red Sea, Polynesia, Southern Japan, and the coasts of Australia [2]. Starry puffer fish usually appears with oval, spherical, or somewhat elongated-shapes. Its skin is prickly rather than scaleless or smooth. The mouth of *A. stellatus* is terminal with four powerful teeth, and the head is massive with a short snout that bears two pairs of nostrils [3]. Except for the ventral body side, the skin is harmoniously dotted with spots. The size of these spots is inversely proportional to the age of the fish [1]. The juveniles have large spots, and the adults have relatively small spots (Fig. 1a-d).

Fish are frequently subjected to the external assaults. Skin, as the largest outer organ of the body, is important in this respect, it not only act as a barrier against external physical, mechanical, biological, and chemical stimuli but also it has an immunological role via limiting the entrance of pathogens into the body [4]. Furthermore, it prevents fish dehydration [5] and performs sensory functions [6]. In some teleost, the skin may have respiratory functions [7, 8].

Fish skin, in contrast to the skin of mammals, is a non-keratinized tegument with living cells. The epidermis is the outermost layer formed mainly by epithelial cells, called keratinocytes, alongside a number of mucus-secreting unicellular glands, and club cells [9, 10]. For adaptation to the aquatic environment, the components of the fish epidermis perform important roles in fish survival and homeostasis [11]. The epidermal keratinocytes are shown to actively react to environmental changes, toxins, and external parasites [4, 12–15]. The epidermal mucus layer is kept from ablation by the highly structured microridges of the surface keratinocytes [16], acts chiefly to trap moisture, inhibit metabolism, and participate in respiration [17]. The next inner layer following the epidermis is dermis which separated from it by an acellular basement membrane. The dermis is thicker, vascularized, formed of two sublayers; the stratum spongiosum and the stratum compactum [18]. Lastly, an internal layer, the hypodermis, is formed by a connective tissue enriched with adipocytes and blood vessels. The latter skin layer is responsible for proper vascularization, pigmentation and mechanical of the whole skin [19–21]. The skin of many fish species has additional features including: scales that get stronger with use [22, 23], and play role in determination age of the fish [24], as well as taste buds that are used for chemoreception [25]. Taste buds, or sensory cells, are considerable secondary epidermal cells that arise between the cells of the superficial layer of the epidermis and have apical microvilli-like sensory hairs [26, 27].

The former fish skin layers undergo changes from embryonic to adult stages. The epidermis maturates from a simple mono- or bi-layered epithelium to a stratified multi-layer epithelium, appearance of specialized dermal appendages such as scales, development of dermal vasculature, and highly expands of the skin area [28, 29].

Few studies have considered potential changes, especially those related to the epidermis, between the skin of juvenile and adult fish which reflect the adaptation of the fish to the surrounding environment. Thus, this study aims to shed light on maturational changes involving the dorso-ventral skin of juvenile starry puffer fish during their transformation into adults using light and scanning electron microscopic analysis. The obtained data have been compared with the previously published research on the skin of other fish species. Further, the future directions of the study will be carried out on the fine structure of the dorso-ventral skin of the juvenile and adult starry puffer fish by using different techniques such as isolating cells and studying cell cycle by flow cytometry and also the transcription level of some immune-relevant genes by RT-PCR.

## Materials and methods

### Study area and fish sampling

Sampling was performed at the front area of the National Institute of Oceanography and Fisheries (Red Sea governorate, Egypt). This area is located approximately about front the shore with 1–2 m of depth, formed by many patches of coral enclosing some sandy areas, seagrass and algae (27◦ 17′ 17′′N and 33◦ 46′ 45′′ E) [30]. Twenty fishes of both sexes (*n* = 10 juveniles and *n* = 10 adults) were collected in 2020.

The starry puffer fishes were caught with gill nets. The net consisted of a single layer and narrow mesh, normally joined together to consist of 10–100 units. The distance between each float piece was 20 cm and a diameter of 5 cm and a thickness of 3 cm. The lead weight was about 16.5 g and the distance between each lead and the following was about 25 cm. The middle layer was a narrow mesh, located between the outer two layers which were characterized by a wider mesh (outer panel). The outer two layers of large mesh netting within which fish will entangle. The float line (float rope) contained a cutting cork (of 6 cm diameter and 4 cm thickness) that the distance between each other of 30 cm and the lead rope (foot rope) found in the lower rope. The distance between each lead and the following about 40 cm and hand 180 cm height.

Fish were euthanized via their exposure to an overdose (250 mg/L) of tricaine methanesulfonate (E10521, Sigma-Aldrich, St. Louis, MO, USA) at a water temperature of 4 °C [31]. Fish death was confirmed two minutes after the last opercular movements and also by the inability of fish to regain active motions in the recovery tank.

### Ethical statement

The research was approved and conducted in accordance with the Animal Ethical Committee's guidelines at the Faculty of Veterinary Medicine, SVU, Qena governoate, Egypt (approval number: VM/SVU/22(1)-04). All methods are reported in accordance with ARRIVE guidelines.

The specimen collection in the current study was complied with the regulations and guidelines of the National Institute of Oceanography and Fisheries, Red sea, Hurghada, Egypt (NIOF-AICUC).

### Scanning electron microscopy

Skin samples representing the dorsal and ventral aspects (*n* = 5 juveniles and *n* = 5 adults) of the middle body part of the freshly collected starry puffer fish were cut, washed in phosphate buffer solution (pH = 7.3), and preserved in 10% neutral buffered formalin [32, 33]. The samples were fixed in a 4% glutaraldehyde solution and postfixed in a 2% buffered osmium tetroxide solution. After being dehydrated and gold-coated, fixed samples were washed in 0.1 M cacodylate buffer. A scanning electron microscope (JSM-4500 LV at 10 kV, JEOL Ltd., Japan) was then used to analyze the specimens.

### Coloring of the scanning electron microscopy (SEM) images

SEM images were coloured using the Photo Filter 7.2.1 application to highlight various structures. Several authors used this technique [34–38].

### CMEIAS color segmentation

**Fig. 2:**
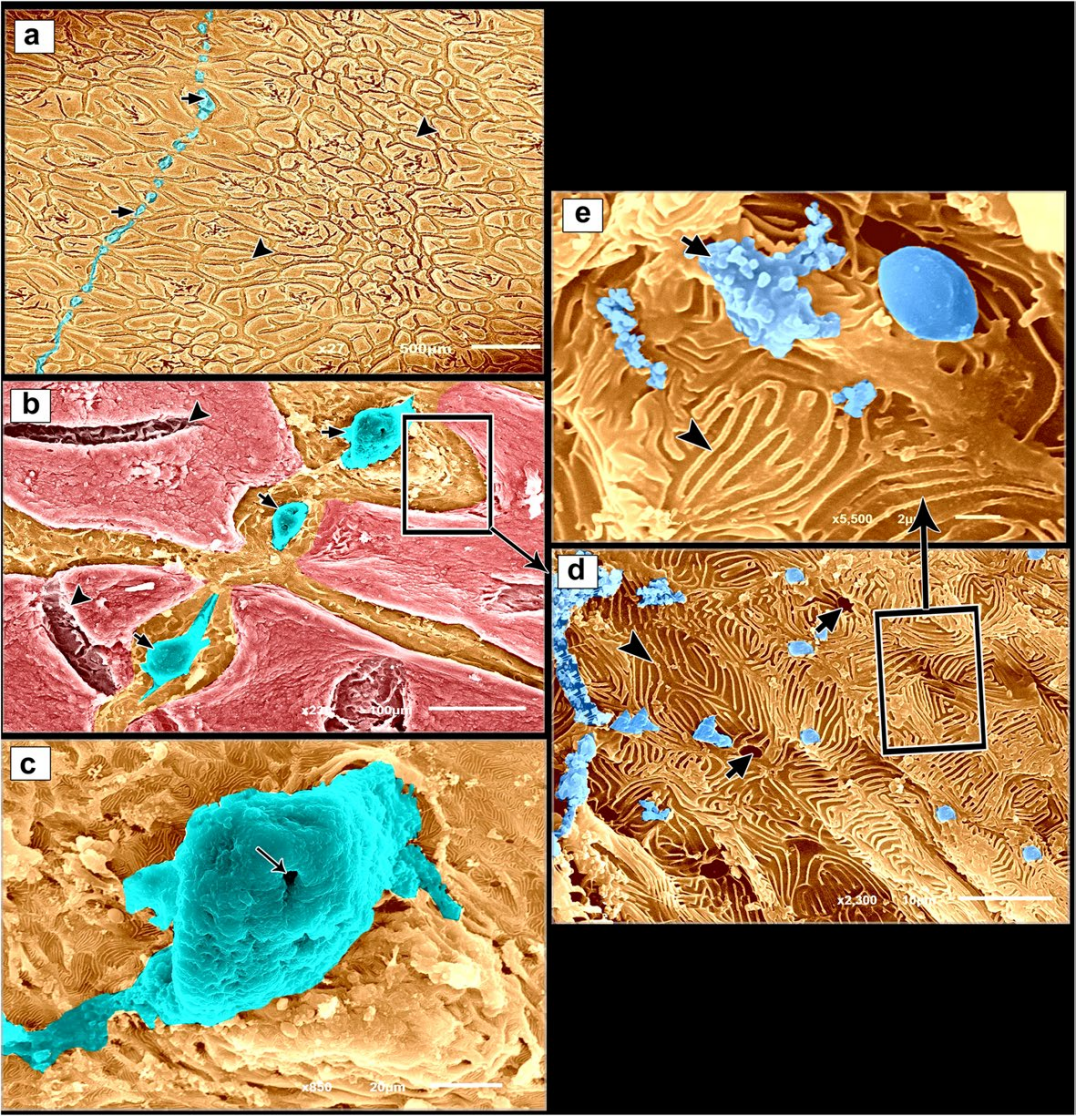
SEM photomicrographs of the dorsal skin of the juvenile starry puffer fish showing: (**a**, **b**) Epidermal surface characterized by the presence of delicate irregular-shaped protrusions with depressed centers (arrowheads), and a row of slightly buried superficial sensory cells (short arrows). **c** Each sensory cell was characterized by the presence of several pores (arrow). **d**, **e** High magnification of the epidermal surface between protrusions. Note the deep epithelial cells (keratinocytes) with compact, extensive, and erratically arranged microridges (arrowheads), and mucous cell openings laden with mucus secretions (short arrows)

The negative images of Figs. 2, 4, and 7 were produced using the CMEIAS colour segmentation and improved computational technology [39]. Several authors engaged in this process [40–42].

### Histology

Skin samples of the dorsal and ventral areas (*n* = 5 juveniles and *n* = 5 adults) were cut into pieces (0.5 cm^3^) and soaked in 10% neutral buffered formalin for 24 h. After proper fixation, the samples were kept in 10% solution of Ethylene Diamine Tetra Acetic Acid (EDTA) for one week until bony tissues softened. The skin samples were dehydrated in ascending grades (70%, 80%, 90%, and 100%) of ethyl alcohol, then cleared in methyl benzoate [43]. Finally, samples were embedded in paraffin wax. Four-μm thick paraffin sections were prepared using Leica RM2235 microtome and stained with H&E and Masson's trichrome in accordance with Suvarna et al. [44].

### Histomorphometric measurements

Histomorphometric measurements of the layers of the dorso-ventral skin of the juvenile and adult starry puffer fish were acquired using Image J software (http://Fiji.sc/Fiji) [45].

### Statistical analysis

The histomorphometric measurements of the present study included the thickness of the epidermis and dermis as well as the diameter of pit organ. All measurements are listed in Table 1 as the means ± standard error (SE), analyzed by SPSS software, version 17.

**Table 1:** Histomorphometric measurements of the dorso-ventral skin in juvenile and adult starry puffer fish

	Juvenile Dorsal skin (M ± SE)	Juvenile Ventral skin (M ± SE)	Adult Dorsal skin (M ± SE)	Adult Ventral skin (M ± SE)
Thickness of Epidermis	17.7 ± 1.3	26.95 ± 1.5	27.654 ± 2.2	45.9 ± 3.06
Thickness of Dermis	599.8 ± 27.6	769.5 ± 24.1	952.3 ± 2.5	781.2 ± 54.7
Pit organ Diameter	303.2 ± 29.1	353.34 ± 23.6	-	293.2 ± 13.45

All measurements (µm) were expressed in mean and standard error (M** ± **SE)

## Results

### Scanning electron microscopy findings

#### Juvenile starry puffer fish

##### Dorsal skin

**Fig. 3:**
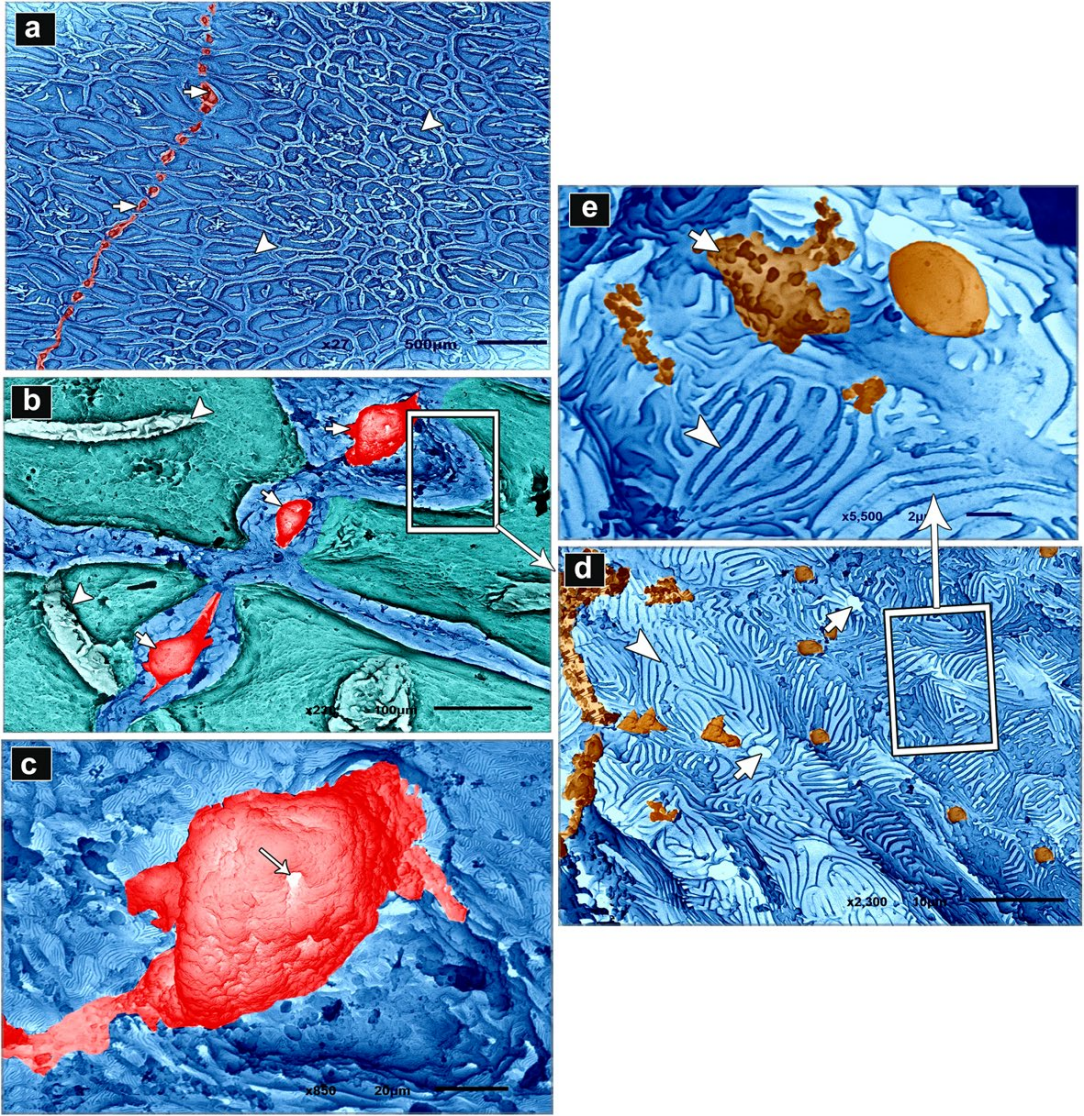
Negative image of Fig. 2 to clarify scanning electron microarchitecture of the dorsal skin of the juvenile starry puffer fish showing: (**a**, **b**) Epidermal surface characterized by the presence of delicate irregular-shaped protrusions with depressed centers (arrowheads), and a row of slightly buried superficial sensory cells (short arrows). **c** Each sensory cell was characterized by the presence of several pores (arrow). **d**, **e** High magnification of the epidermal surface between protrusions. Note the deep epithelial cells (keratinocytes) with compact, extensive, and erratically arranged microridges (arrowheads), and mucous cell openings laden with mucus secretions (short arrows)

**Fig. 4:**
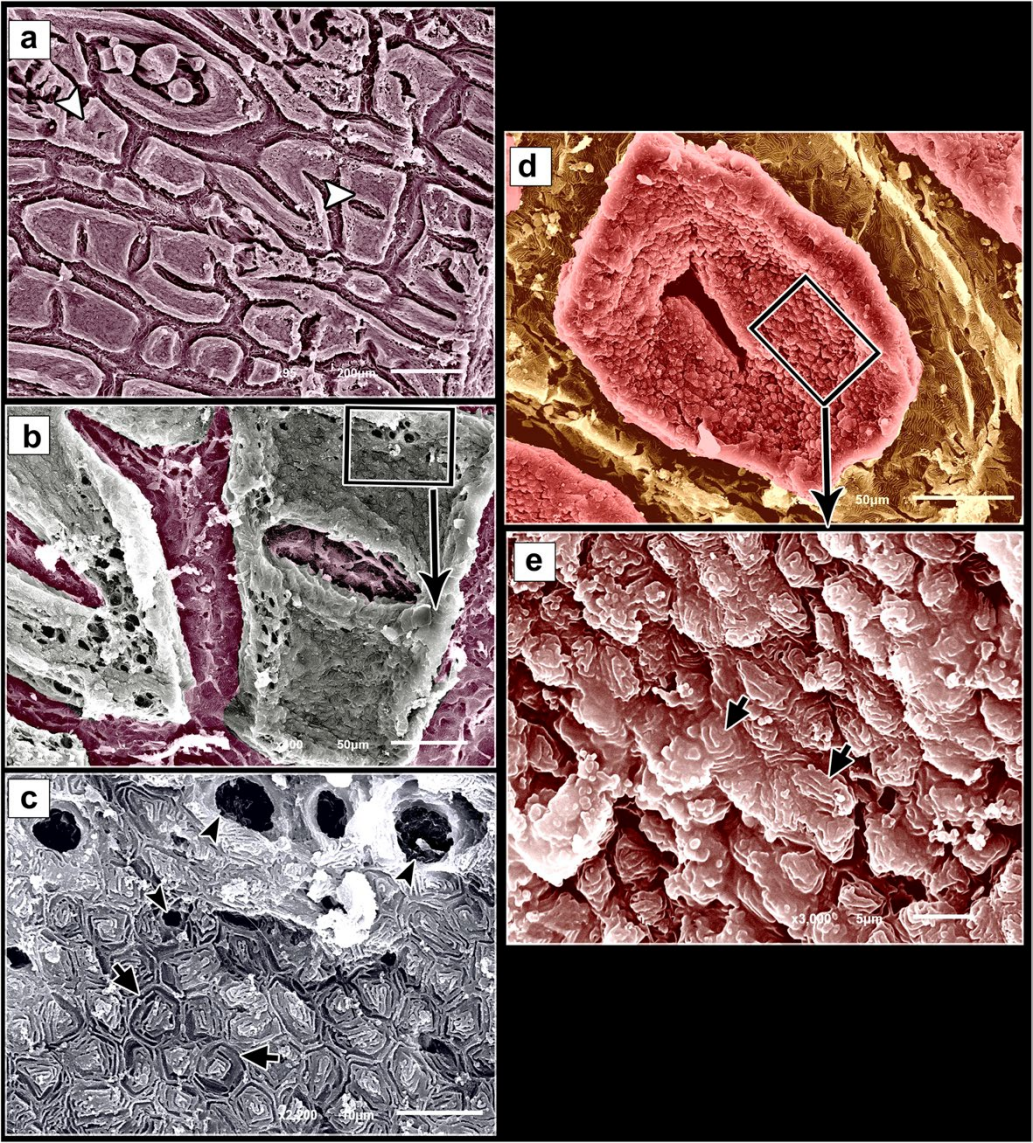
SEM photomicrographs of the dorsal skin of the juvenile starry puffer fish showing: (**a**, **b**) Some rectangular, square-shaped protrusions were observed on the epidermal surface. **c** High magnification of the edges of rectangular-like protrusions of the epidermis. Note variable sizes of the mucous cell openings (arrowheads), and adjacent superficial epithelial cells were demarcated from each other by low borders like ridges (short arrows). **d**, **e** Some superficial epithelial cells revealed indistinct outlines with ill-defined and interrupted microridges (short arrows)

The epidermal surface of the dorsal skin of the juvenile starry puffer fish was characterized by delicate irregular-shaped protrusions, some of which had depressions. A few of the protrusions taken were rectangular and squared-shaped (Figs. 2a, b; 3a, b; 4a, b, d; and 5a, b, d). Superficial sensory cells, buried slightly in the epidermis, were arranged in rows. Each sensory cell was dome-shaped, characterized by the presence of several pores (Figs. 2a-c and 3a-c).

The epidermal surface between the protrusions was represented by deep epithelial cells (keratinocytes) with compact, extensive, and erratically arranged microridges, and few mucous cell openings laden with mucous secretions (Figs. 2d, e and 3d, e).

**Fig. 5:**
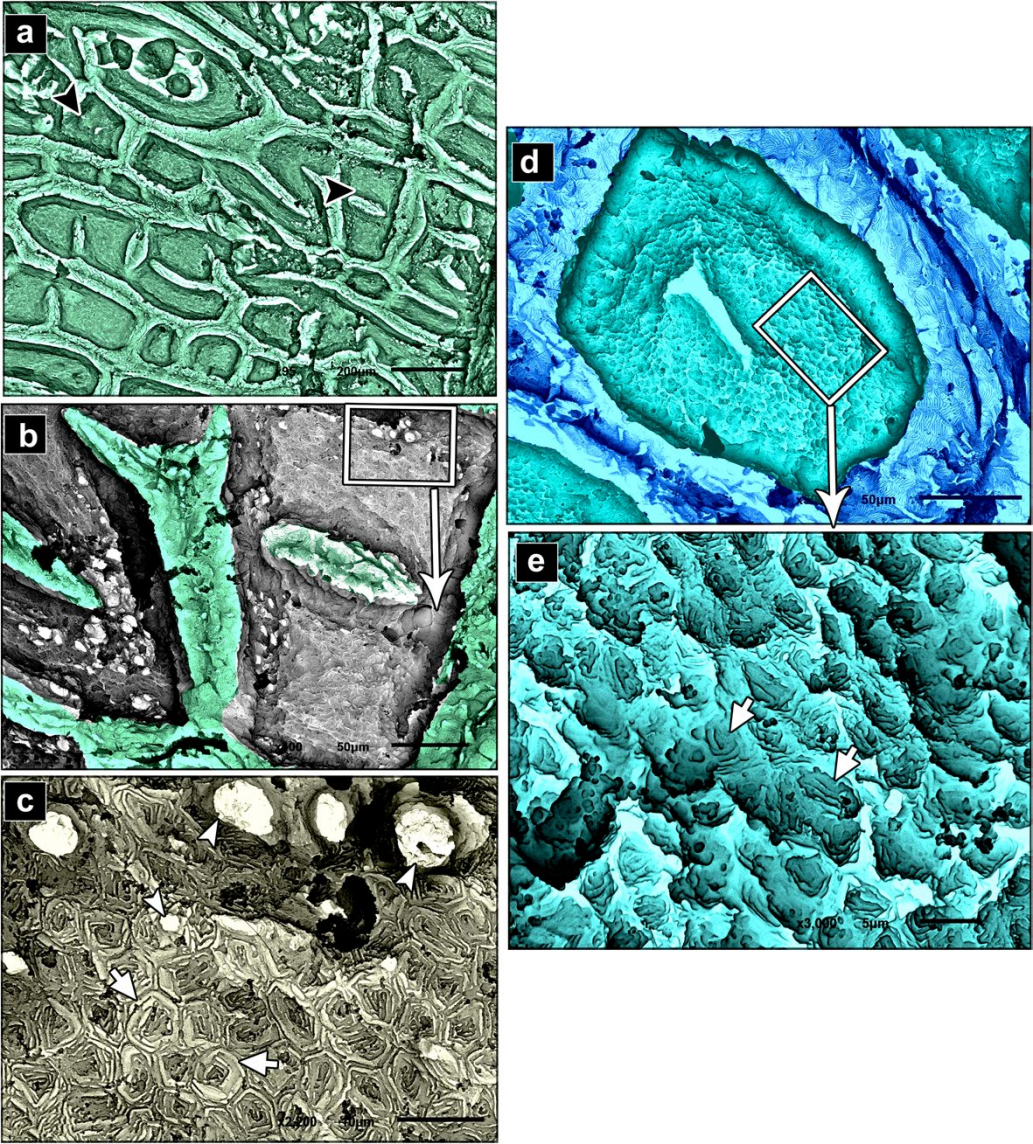
Negative image of Fig. 4 to clarify scanning electron microarchitecture of the dorsal skin of the juvenile starry puffer fish showing: (**a**, **b**) Some rectangular, square-shaped protrusions were observed on the epidermal surface. **c** High magnification of the edges of rectangular-like protrusions of the epidermis. Note, variable sizes of the mucous cell openings (arrowheads), and adjacent superficial epithelial cells were demarcated from each other by low borders like ridges (short arrows). **d**, **e** Some superficial epithelial cells revealed indistinct outlines with ill-defined and interrupted microridges (short arrows)

Variable sized mucous cell openings were observed on the edges of the rectangular-like protrusions of the epidermis (Figs. 4b, c and 5b, c). Additionally, some adjacent superficial epithelial cells with short and interrupted microridges were demarcated from each other by low border like ridges (Figs. 4c and 5c). While, the others were indistinct with ill-defined microridges (Figs. 4d, e and 5d, e).

##### Ventral skin

**Fig. 6:**
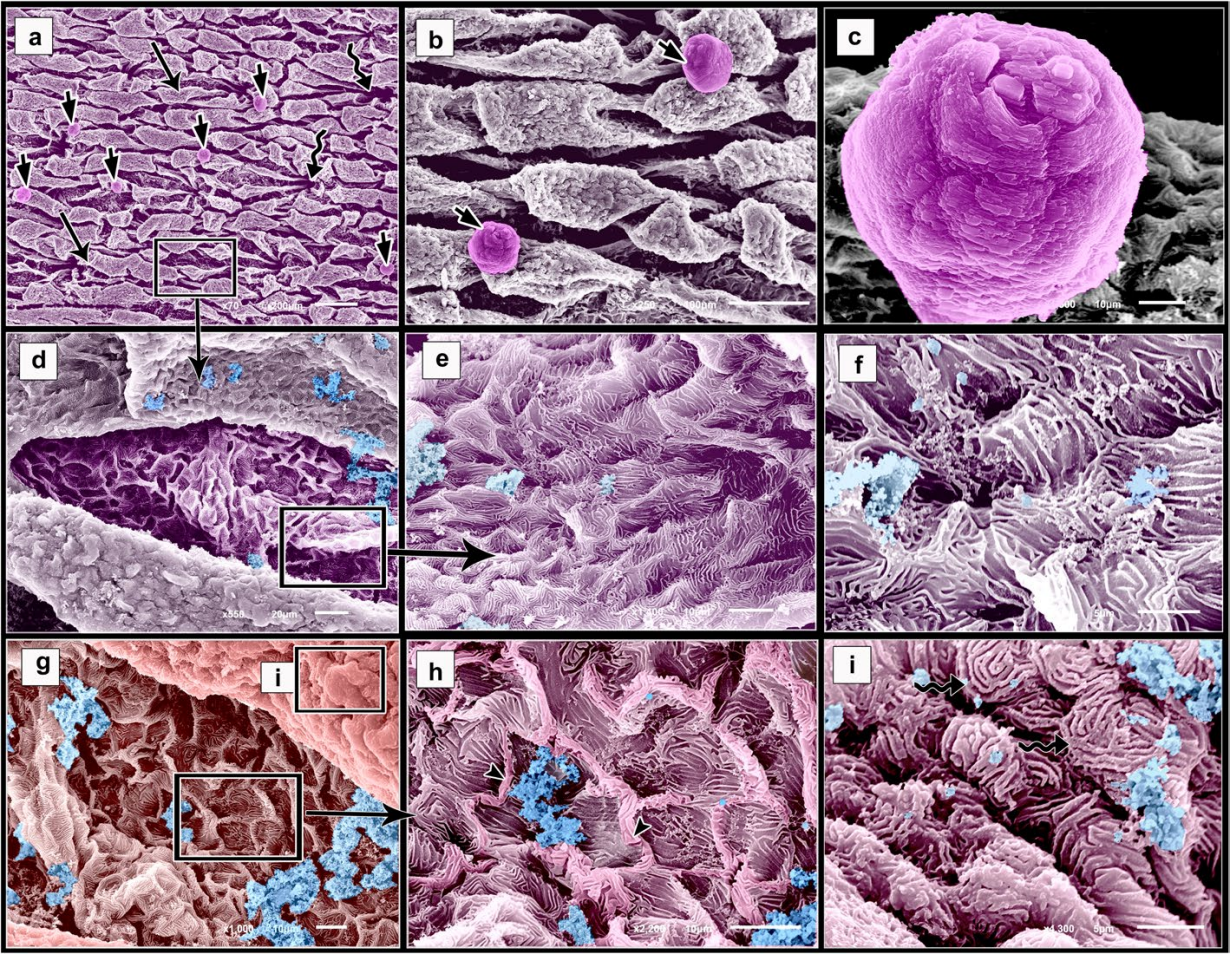
SEM photomicrographs of the ventral skin of the juvenile starry puffer fish showing: (**a**-**c**) The epidermal surface was represented by interrupted folds (long arrows), openings of the mucous glands (twisted arrows), several symmetrically-sized sensory cells (short arrows). **d**-**f** The epidermal surface between the folds was discernible in deep epithelial cells with compact, extensive, and erratically arranged microridges. **g**-**i** The epidermal surface of the folds and depressions in between (**g**). Note in (**h**) the adjacent deep epithelial cells were demarcated from each other by well-distinct borders like ridges (arrowheads) and in (**i**) the superficial epithelial cells with concentrically organized microridges (twisted arrows)

The epidermal surface of the ventral skin of the juvenile starry puffer fish was characterized by interrupted folds. On the surface of these folds, abundant symmetrically-sized sensory cells were irregularly distributed with the irregular outline openings of the mucous glands (Fig. 6a-c). The epidermal surface of the depressions between the folds had some deep epithelial cells with ill-distinct compact, extensive, and erratically arranged microridges (Fig. 6d-f), and the other adjacent deep epithelial cells were demarcated from each other by well-distinct border like ridges (Fig. 6g, h). On the other hand, the epidermal surface of the folds had superficial epithelial cells with concentrically organized microridges (Fig. 6i).

#### Adult starry puffer fish

##### Dorsal skin

**Fig. 7:**
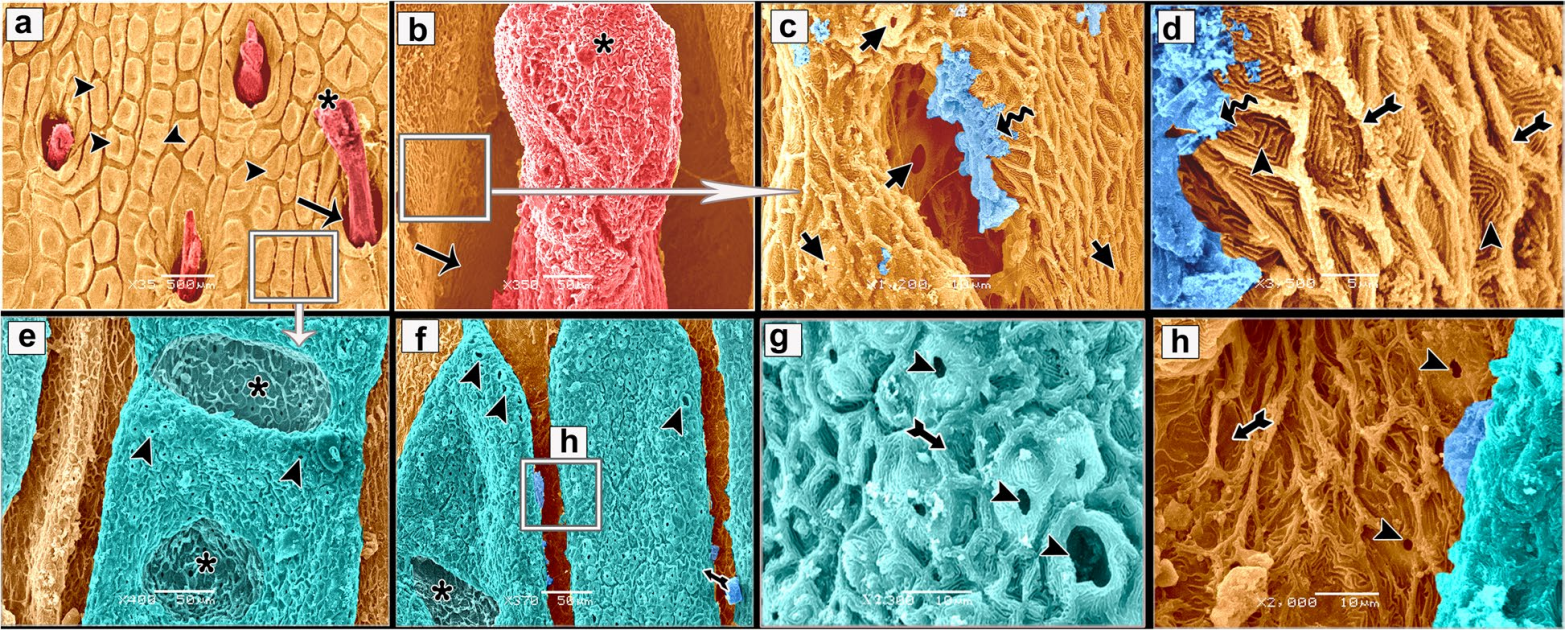
SEM photomicrographs of the dorsal skin of the adult starry puffer fish showing: (**a**, **b**) Epidermal surface characterized by the presence of variable sized and shaped bricks-like elevations (arrowheads) arranged in a longitudinal manner, and odontic spines of variable heights (asterisks) emerging from hole-like sinuses (arrows). **c**, **d** High magnifications of the inner epithelial surface of the sinus. Note mucous cell openings (short arrows) with mucous secretions (twisted arrows), polygonal deep epithelial cells (keratinocytes) with extensive concentrically organized microridges (arrowheads) and distinct borders like ridges (barbed arrows). **e**, **f** Bricks-like elevations of the epidermis had variable shapes depressions (asterisks), characterized by the presence of abundant mucous cell openings that appeared as ant house colonies (arrowheads), and the superficial epithelial cells (barbed arrow). **g** High magnification of the edges of bricks-like elevations of the epidermis. Note the round to oval-shaped mucous cell openings (arrowheads), and the superficial epithelial cells (barbed arrow). **h** High magnification of the epidermal surface between elevations. Note the few mucous cell openings (arrowheads), and the deep epithelial cells with reduced microridges (barbed arrow)

**Fig. 8:**
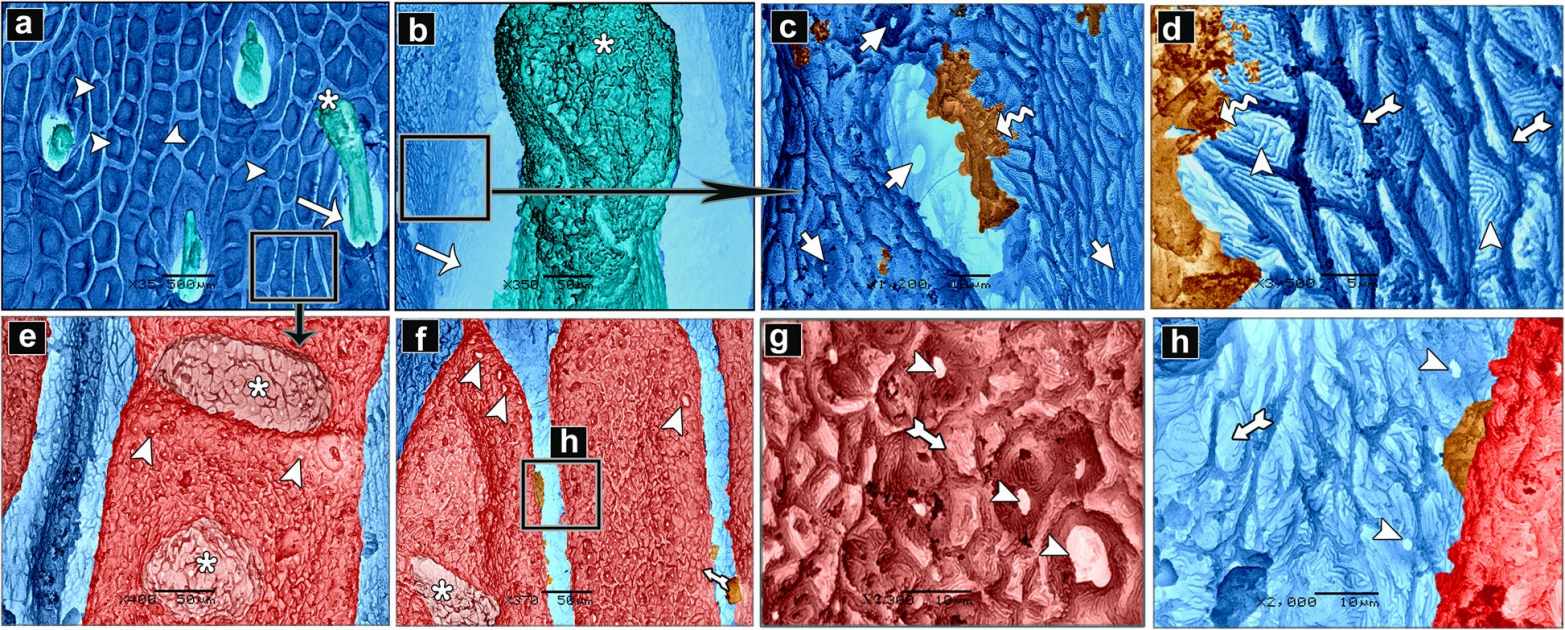
Negative image of Fig. 7 to clarify scanning electron microarchitecture of the dorsal skin of the adult starry puffer fish showing: (**a**, **b**) Epidermal surface characterized by the presence of variable sized and shaped bricks-like elevations (arrowheads) arranged in a longitudinal manner, and odontic spines of variable heights (asterisks) emerging from hole-like sinuses (arrows). **c**, **d** High magnifications of the inner epithelial surface of the sinus. Note mucous cell openings (short arrows) with mucous secretions (twisted arrows), polygonal deep epithelial cells (keratinocytes) with extensive concentrically organized microridges (arrowheads) and distinct borders like ridges (barbed arrows). **e**, **f** Bricks-like elevations of the epidermis had variable shapes depressions (asterisks), characterized by the presence of abundant mucous cell openings that appeared as ant house colonies (arrowheads), and the superficial epithelial cells (barbed arrow). **g** High magnification of the edges of bricks-like elevations of the epidermis. Note the round to oval-shaped mucous cell openings (arrowheads), and the superficial epithelial cells (barbed arrow). **h** High magnification of the epidermal surface between elevations. Note the few mucous cell openings (arrowheads), and the deep epithelial cells with reduced microridges (barbed arrow)

The epidermal surface of the dorsal skin of the adult starry puffer fish was distinguished by variable sized and shaped bricks-like elevations (rectangular, square, triangular, elliptical-shaped), and variable heighted odontic spines with pointed and blunt apices. The epidermal elevations were arranged in a longitudinal manner and the spines emerged from hole-like sinuses (Figs. 7a, b & 8 a, b). By the high magnifications, the inner epithelial surface of the sinus was characterized by the presence of a few mucous cell openings with mucous secretions and deep epithelial cells (keratinocytes). The deep epithelial cells were large polygonal; their surfaces were distinguished by extensive concentrically organized microridges. These microridges were smooth and unbranched. The adjacent deep epithelial cells were demarcated from each other by distinct thick border like ridges giving a honeycomb appearance and covered by excessive mucous (Figs. 7c, d and 8c, d).

Most of the epidermal surface of the bricks-like elevations had variable shapes depressions (Figs. 7e, f and 8e, f). Furthermore, this surface was characterized by the presence of abundant round, oval-shaped mucous cell openings which appeared as ant house colonies and small superficial epithelial cells. The large mucous cell openings were mainly observed on the edges of the elevations (Figs. 7e-g and 8e-g). The superficial epithelial cells resembled the former deep epithelial cells but they were smaller with less defined microridges (Figs. 7g and 8g). On the other hand, the epidermal surface between the elevations was discernible in few mucous cell openings and some deep epithelial cells with reduced microridges (Figs. 7h and 8h).

##### Ventral skin

**Fig. 9:**
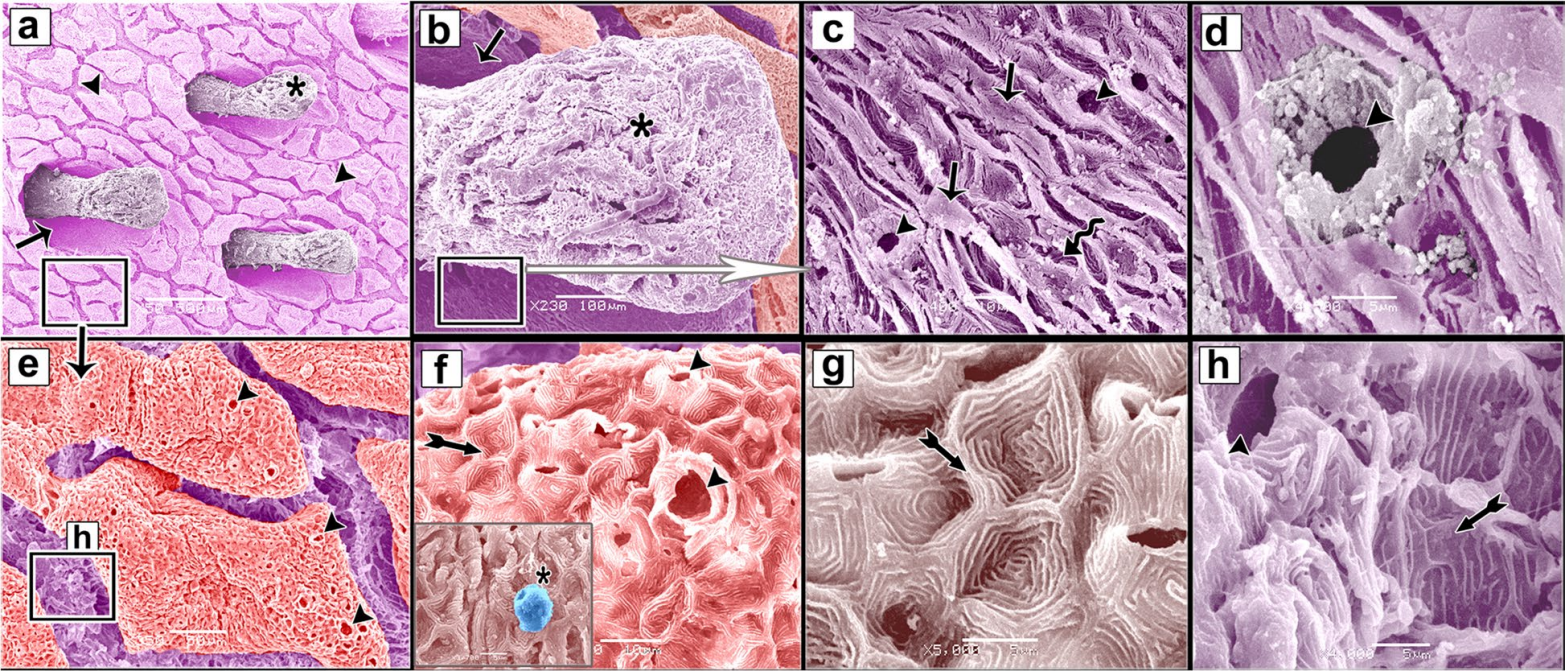
SEM photomicrographs of the ventral skin of the adult starry puffer fish showing: (**a**, **b**) Epidermal surface characterized by the presence of irregular-shaped protrusions (arrowheads) arranged in an erratic manner, odontic spines of symmetric heights (asterisks) emerging from hole-like sinuses (arrows). **c**, **d** High magnifications of the inner epithelial surface of the sinus. Note the corrugated folds (arrows), the mucous cell openings (arrowheads) with blobs of mucous on their margins, and the ill-defined deep epithelial cells (twisted arrows). **e**, **f** The epidermal surface of the irregular-shaped elevations was characterized by the presence of abundant mucous cell openings (arrowheads), superficial epithelial cells (barbed arrow), and a sensory cell (asterisk). **g** High magnification of the superficial epithelial cells (barbed arrow). Note the extensive concentrically organized microridges with ill-defined borders like ridges approximately as the fingerprint pattern. **h** High magnification of the epidermal surface between the elevations. Note the less frequently seen large mucous cell openings (arrowhead), and the deep epithelial cells with reduced or short microridges (barbed arrow)

The epidermal surface of the ventral skin of the adult starry puffer fish was marked by irregular-shaped protrusions (arranged in an erratic manner) and symmetrically height odontic spines within the sinuses which were larger than those observed at the dorsal skin (Fig. 9a, b). Some areas of the epidermal surface of the ventral skin appeared smooth free from any protrusions. In the high magnification, the inner epithelial surface of the sinuses was characterized by corrugated folds, mucous cell openings with blobs of mucous selections on their margins, and ill-defined deep epithelial cells (Fig. 9c, d).

The epidermal surface of the irregular-shaped protrusions had abundant mucous cell openings, superficial epithelial cells as those at the dorsal surface as well as sensory cells were observed (Fig. 9e, f). By the high magnification, the superficial epithelial cells were distinguished by extensive concentrically organized microridges with ill-defined borders like ridges which gave fingerprints-like appearance (Fig. 9g). The characterization of the epidermal surface between the protrusions was as that mentioned in the dorsal skin, but the openings of the mucous cells were larger (Fig. 9h).

### Histological findings

#### Juvenile starry puffer fish

##### Dorsal skin

**Fig. 10:**
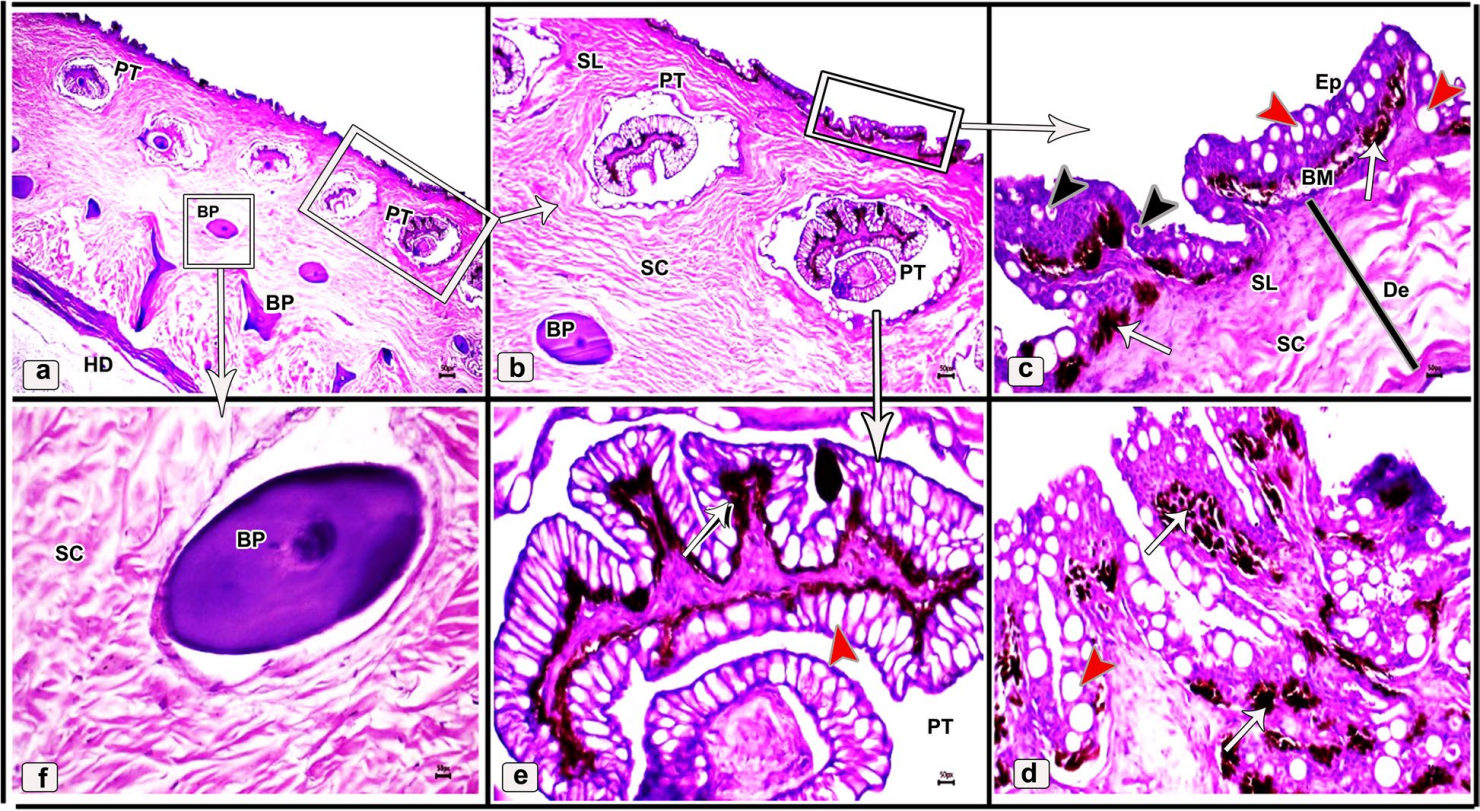
Histological micrographs of the dorsal surface skin of the juvenile starry puffer fish showing: Epidermis (Ep), basement membrane (BM), dermis (De) with stratum laxum (SL) and stratum compactum (SC), club cell (red arrowheads), mucous cell (black arrowheads), pit organ (PT), bony plate (BP), hypodermis (HD), and chromatophores (white arrows). H&E stain. Magnifications are X20 (**a**), X40 (**b**), X100 (**c**, **f**), and X400 (**d**, **e**)

Histologically, the dorsal skin surface of the Juvenile starry puffer fish comprised three main layers: the epidermis, dermis, and hypodermis. The epidermis was lined by free-keratinized stratified squamous epithelium with club cells and mucous cells all resting on a thick, wavy basement membrane (Fig. 10a-c). The club cells were large spherical cells that occupied most cells of the epidermal layer, while the mucous cells were located along the superficial epidermal layer and full of viscous secretions (Fig. 10C). The dermal layer was obviously differentiated into two principal layers: outer stratum laxum and inner stratum compactum layer. The stratum laxum formed of a loose connective tissue layer beneath the basement membrane (Fig. 10b, c). In addition, numerous melanophores with rough brown to black granules were observed (Fig. 10c). Stratum compactum layer was represented by a fibrous connective tissue layer, embracing variable shapes and sizes of bony plates (Fig. 10a-d and f). Moreover, there were abundant large spherical pit organs between the two dermal layers; laxum and compactum. The pits had large number of club cells, melanophores and encircled with pericellular ring (Fig. 10a-b and d-e).

##### Ventral skin

**Fig. 11:**
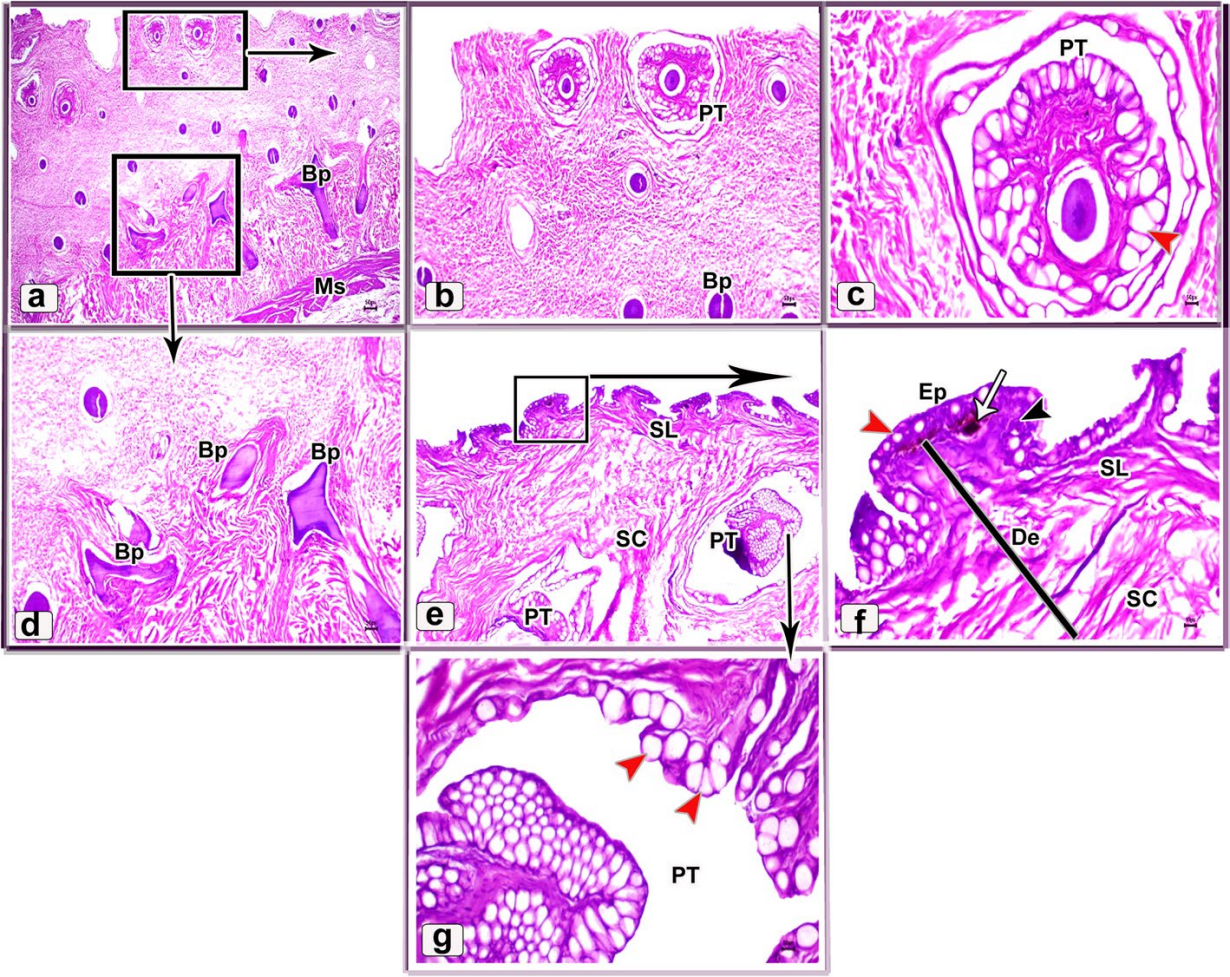
Histological micrographs of the ventral surface skin of the juvenile starry puffer fish showing:  Epidermis (Ep), dermis (De) with stratum laxum (SL) and stratum compactum (SC), club cell (red arrowheads), mucous cell (black arrowheads), pit organ (PT), bony plate (BP), chromatophores (white arrows), and muscle fibers (Ms). H&E stain. Magnifications are X10 (**a**), X40 (**b**, **d**, **e**), X100 (**c**, **f**), and X400 (**g**)

The ventral surface of the juvenile starry puffer fish displayed similar histological features to that of the dorsal skin side but, the pit organs were noticed close proximity to the superficial epidermal layer and some of them were supported by bony plates (Fig. 11a-d and g). Additionally, also few melanophores were located along the dermal laxumal layer if compared to the dorsal skin region (Fig. 11e, f).

**Fig. 12:**
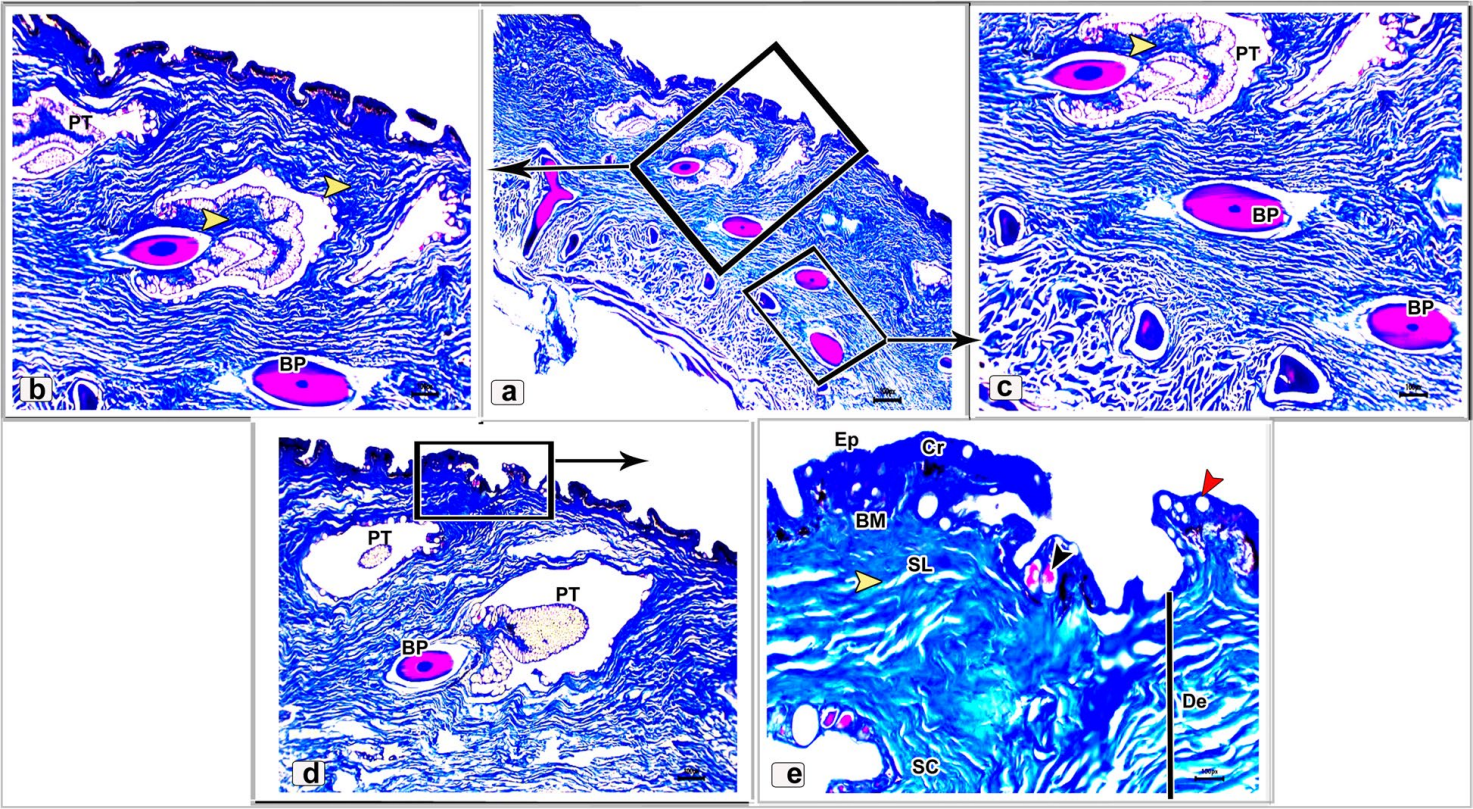
Histological micrographs of the dorsal surface skin of the juvenile starry puffer fish showing:  Distribution of the collagen fibers (yellow arrowheads), epidermis (Ep), basement membrane (BM), dermis (De) with stratum laxum (SL) and stratum compactum (SC), club cell (red arrowheads), mucous cell (black arrowheads), pit organ (PT), bony plate (BP), and chromatophores (Cr). Masson's trichrome stain. Magnifications are X20 (**a**), X40 (**b**, **c**, **d**), and X100 (**e**)

**Fig. 13:**
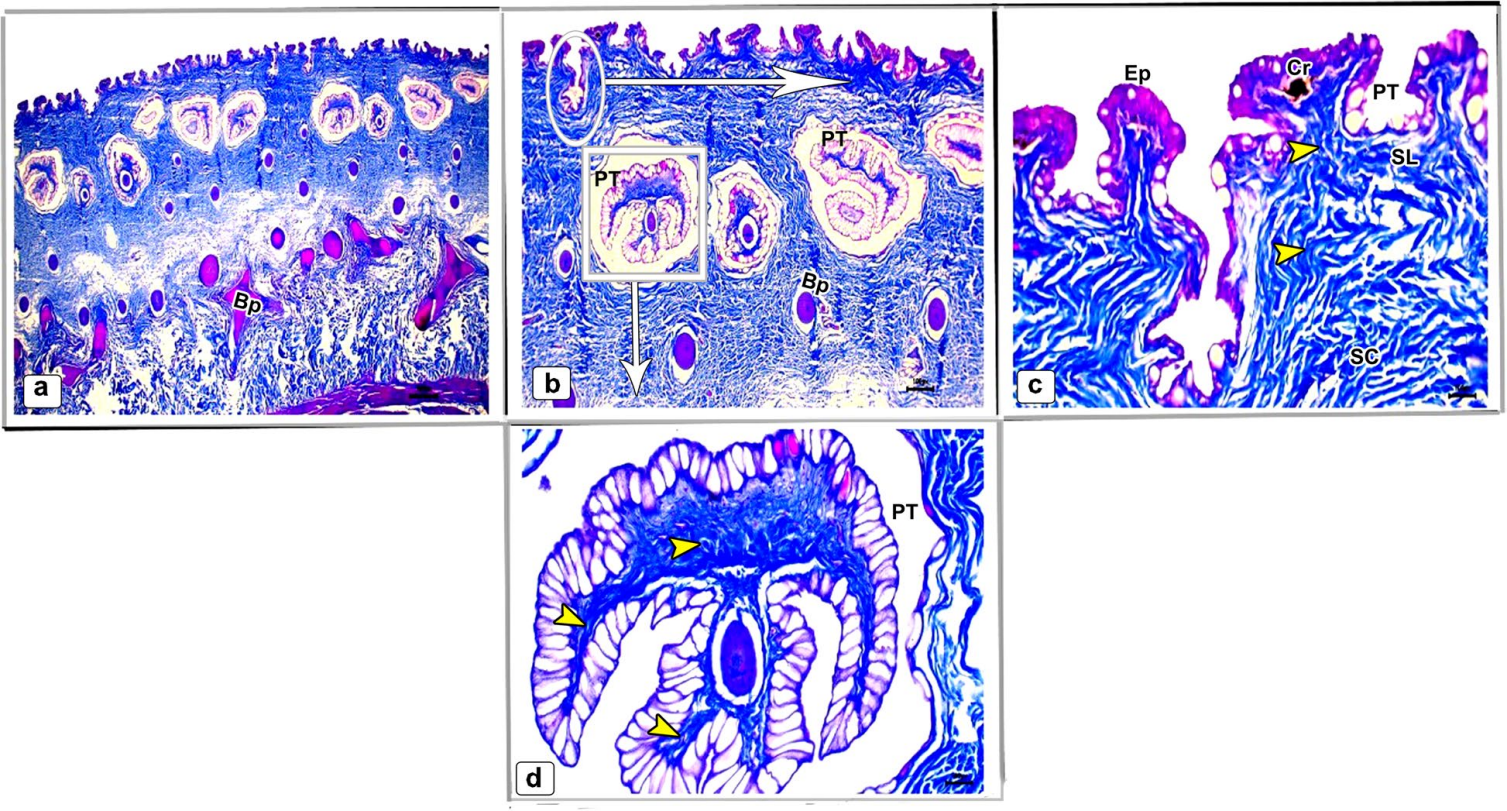
Histological micrographs of the ventral surface skin of the juvenile starry puffer fish showing: Distribution of the collagen fibers (yellow arrowheads), epidermis (Ep), dermis (De) with stratum laxum (SL) and stratum compactum (SC), pit organ (PT), bony plate (BP), and chromatophores (Cr). Masson's trichrome stain. Magnifications are X20 (**a**), X40 (**b**), X100 (**c**), and X400(**d**)

In the skin of the dorsal and ventral sides of the juvenile starry puffer fish, collagenous fiber bundles were observed in packed and parallel groups. These fibers were scattered within the dermal skin layer and also occupied the spaces between the pit organs and bony plates (Figs. 12a-e and 13a-d).

#### Adult starry puffer fish

##### Dorsal skin

**Fig. 14:**
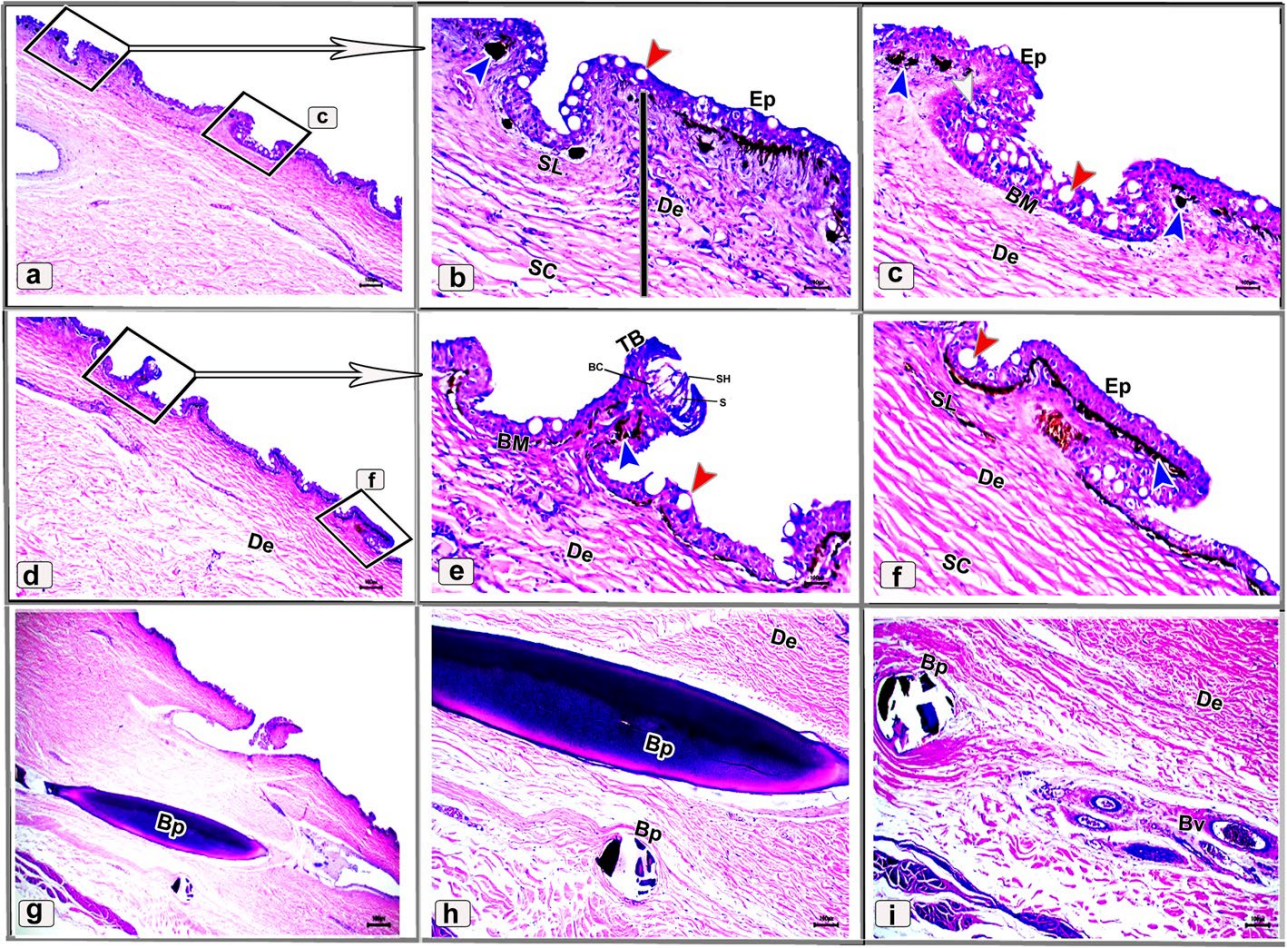
Histological micrographs of the dorsal surface skin of the adult starry puffer fish showing: Epidermis (Ep), basement membrane (BM), dermis (De) with stratum laxum (SL) and stratum compactum (SC), club cell (red arrowheads), mucous cell (white arrowheads, bony plate (BP), taste buds (TB), basal cells (Bc), supporting cells (S), sensory hairs (SH), chromatophores (blue arrowheads), and blood vessels (BV). H&E stain. Magnifications are X40 (**a**, **d**, **g**), and X100 (**b**, **c**, **e**, **f**, **h**, **i**)

The histological observations of the dorsal skin of the adult starry puffer fish revealed two large parts, the epidermis and dermis (Fig. 14a-f). At higher magnified sections, the epidermal layer lined by non-cornified stratified squamous epithelium included a single row of simple columnar cells covering the thick basement membrane and followed by multilayer cells interspersed by large, rounded club cells and mucous cells. The outermost layer of the epidermis was covered by flat and un-cornified squamous cells. Under the basement membrane, the melanophore pigments were observed as disconnected black pigment granules (Fig. 14b, c and e, f). Moreover, few taste buds were noticed at the superficial epidermal cell layer. Each taste bud was formed of basal cells, supporting cells, and sensory hair cells (Fig. 14d, e). The second skin layer (dermis) had the outer stratum laxum and inner stratum compactum. The compactum layer was made up of connective tissue and housed several bony plates and blood vessels as well (Fig. 14f-i).

##### Ventral skin

**Fig. 15:**
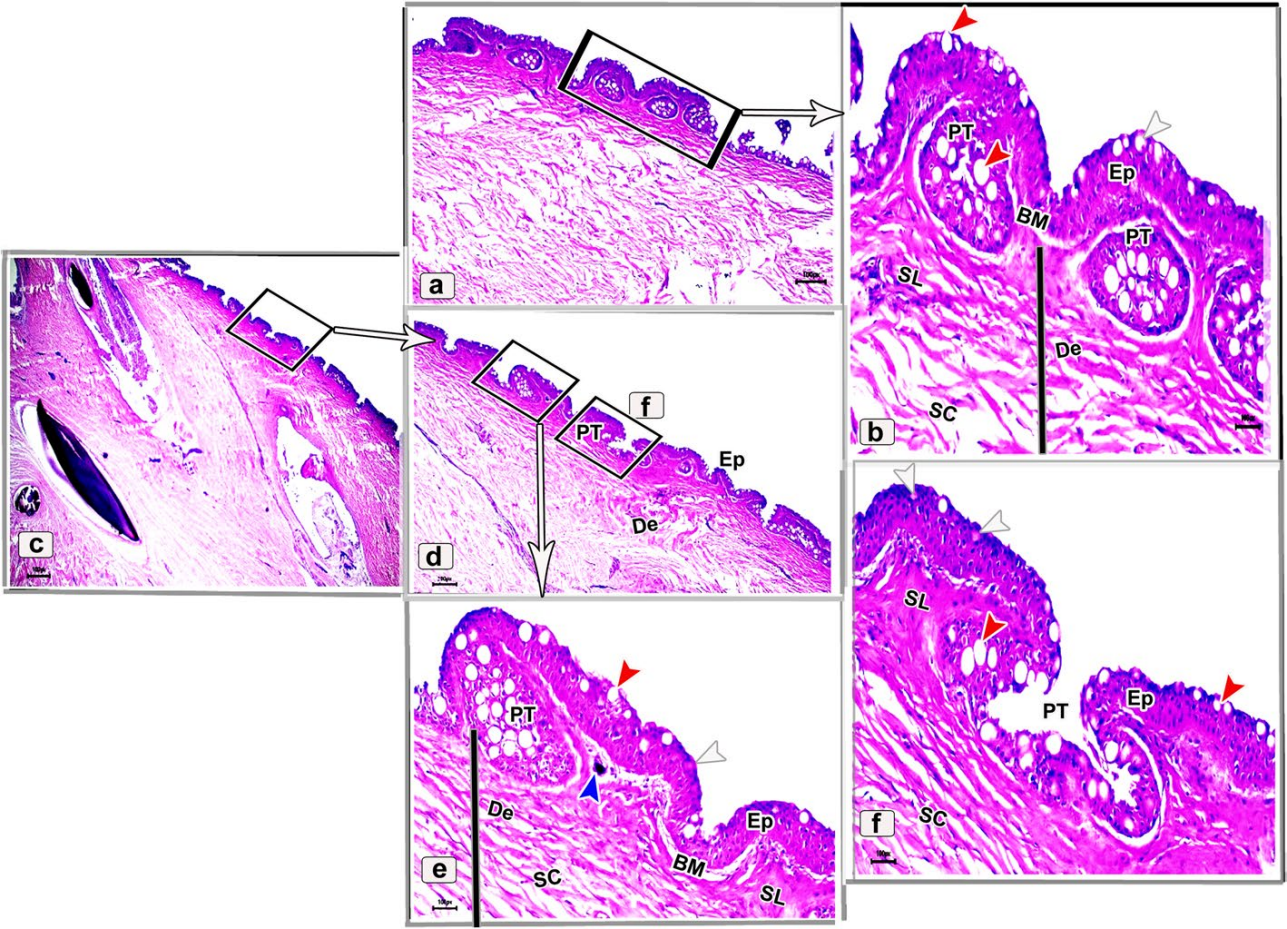
Histological micrographs of the ventral surface skin of the adult starry puffer fish showing: Epidermis (Ep), basement membrane (BM), dermis (De) with stratum laxum (SL) and stratum compactum (SC), club cell (red arrowheads), mucous cell (white arrowheads), bony plate (BP), pit organ (PT), and chromatophores (blue arrows). H&E stain. Magnifications are X20 (**c**), X40 (**a**, **d**), X100 (**b**, **e**), and X400 (**f**)

The histological investigations of the ventral side skin of the starry puffer fish showed the two basic skin layers, the outer epidermis and inner dermis layer and revealed the same features noticed in the dorsal side skin (Fig. 15a-f). Few melanophores were detected underneath the basement membrane (Fig. 15d, e). Furthermore, several pit organs were present below the basement membrane and also were noticed in between the epidermal cells (Fig. 15a, b and d-f). In addition, bony plates were also present in the deep dermal layer (Fig. 15c).

**Fig. 16:**
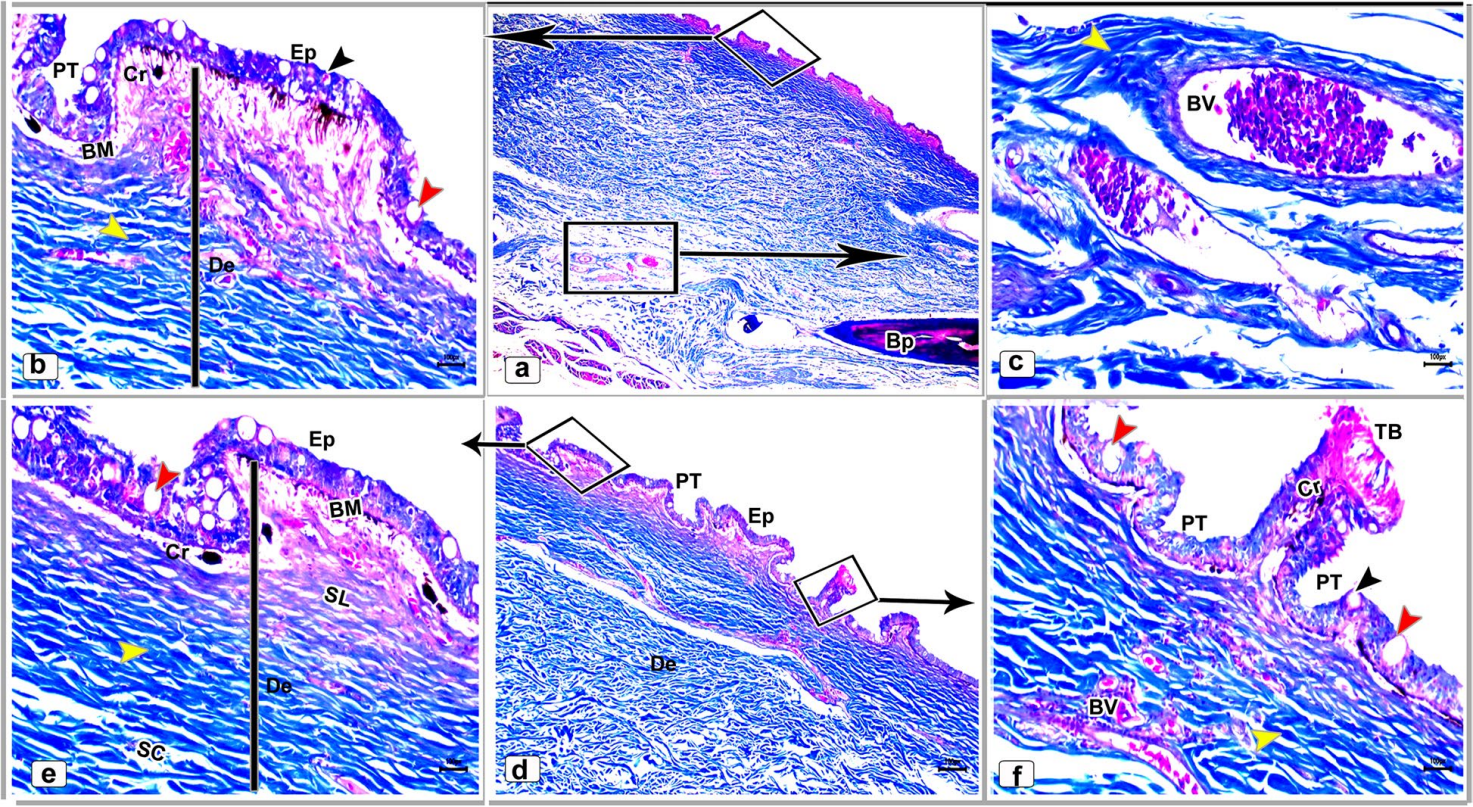
Histological micrographs of the dorsal surface skin of the adult starry puffer fish showing: Distribution of the collagen fibers (yellow arrowheads), epidermis (Ep), basement membrane (BM), taste bud (TB), dermis (De) with stratum laxum (SL) and stratum compactum (SC), club cell (red arrowheads), mucous cell (black arrowheads), pit organ (PT), bony plate (BP), chromatophores (Cr), and blood vessels (BV). Masson's trichrome stain. Magnifications are X20 (**a**), X40 (**d**), X100 (**b**, **e**, **f**), and X400 (**c**)

**Fig. 17:**
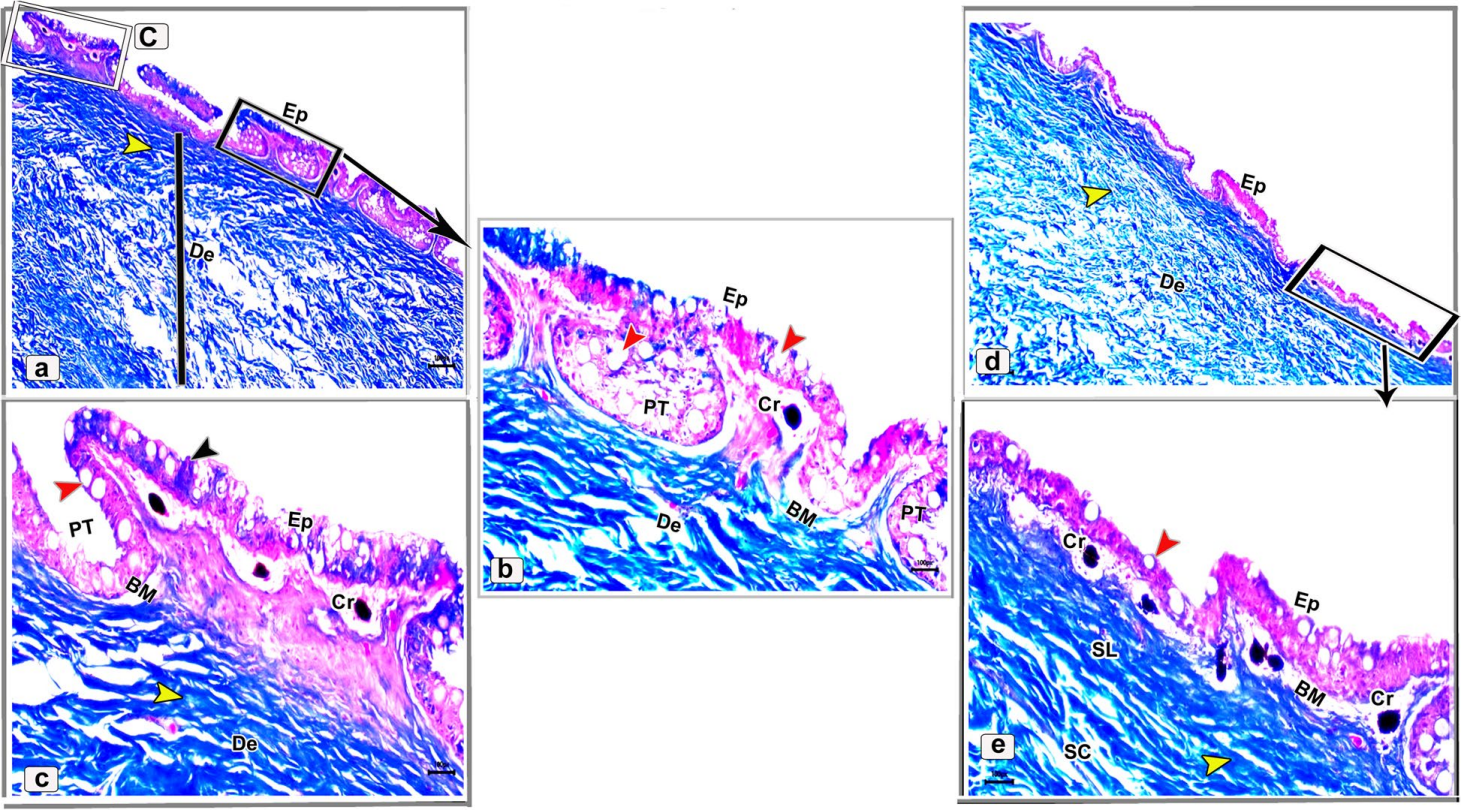
Histological micrographs of the ventral surface skin of the adult starry puffer fish showing: Distribution of the collagen fibers (yellow arrowheads), epidermis (Ep), basement membrane (BM), dermis (De) with stratum laxum (SL) and stratum compactum (SC), club cell (red arrowheads), mucous cell (black arrowheads), pit organ (PT), and chromatophores (Cr). Masson's trichrome stain. Magnifications are X20 (**d**), X40(**a**), X100 (**b**, **e**), and X400(**c**)

A well-developed connective tissue layer rich in collagen fibers was observed within the dermal layer of both dorsal and ventral sides skin in the adult starry puffer fish (Figs. 16a-f and 17a-e).

### Histomorphometric findings

As shown in Table 1, the investigated histological data demonstrated a marked difference in the thickness of both epidermal and dermal skin layers in both juvenile and adult starry puffer fishes. The highest epidermal thickness (45.9 ± 3.06) was recorded in the ventral side skin of the adult fish, while the lowest thickness (17.7 ± 1.3) was in the dorsal side skin of the juvenile. Meanwhile, the highest dermal skin thickness (952.3 ± 2.5) was noticed in the dorsal side skin of the adult fish and the lowest thickness (599.8 ± 27.6) was in the dorsal side skin of the juvenile fish. In addition, our analyzed histological data described that the diameter of the pit organ reached its highest value (353.34 ± 23.6) in the ventral side skin of the juvenile fish, while the lowest value was reported in the ventral side skin of the adult one (293.2 ± 13.45).

## Discussion

The present study involved the histological and ultrastructural characteristics of the skin in the starry pufferfish (*Arothron stellatus*, Anonymous 1798). The studied species is a highly poisonous tetradontidae fish, particularly its skin, viscera, and blood. The skin has a significant role in the inflation of fish body during a poisonous response. Starry pufferfish are present in large numbers in the Indo-Pacific region. In spite of the poisonous nature of the fish, their meat stands as one of the most delicious and expensive dishes in Japan and other Asian countries.

Generally, fish skin is supposedly the toughest organ since it protects its integrity [4]. It differs from that of other exposed vertebrates where, the epidermis comes into touch with the outside environment. Fish skin is exposed to many stresses as a result of its aquatic habitat; osmotic pressure (between epidermal cells and H_2_O) [46] and the physical forces (from water and other environmental dangers such as stones, rocks, and coral reefs). Additionally, the skin is also easily accessible to disease-causing organisms including fungi, bacteria, and parasites [47]. The adaptations to the former stresses differ among different fish species and stages. Inasmuch, data for the study of the skin of the juvenile and the adult fish are scarce, so, the study in question discusses the histo-ultrastructural investigations of the dorsal–ventral skin of the starry puffer fish.

Interestingly, fish scales are a basic component of fish skin and act as armor to defend the animal from predators, improve swimming, and act as mineral reservoirs [22, 48–50]. Ytteborg et al. [23] reported that polar fish is characterized by a folded outer surface with interrupted scales and Atlantic cod has a smooth epidermal surface and overlapping scales. However, our scanning electron microarchitecture of the dorso-ventral skin of the juvenile and the adult fish revealed scalelss skin. Delicate irregular-shaped protrusions and well-defined bricks-like elevations were demonstrated in the dorsal skin, while the ventral skin had interrupted folds, and irregular-shaped protrusions in the juvenile and the adult fish, respectively. The folded skin characterizations of the current studied species enable inflating their bodies by swallowing air or water which assists the fishes to ward off potential enemies. Moreover, Noguchi and Arakawa [51] stated functionally that the skin, ovaries, and liver of the starry puffer fish *(Arothron stellatus)* contain a lethal poison (Tetrodotoxin), which serves as the species' defense against ravenous predators. It becomes toxic because it consumes bacteria that contain the toxin.

The epithelial cells (keratinocytes) in the epidermis display elevated ridges termed microridges [52]. Cordero et al. [53] support our investigations in adult fish, the area of the superficial and deep epithelial cells (keratinocytes) in the dorsal skin were occupied by larger and well-defined microridges than in the ventral skin, contrary was seen in the juvenile fish skin. Furthermore, in the present study, as well as those of Collin and Collin [54] and Hawkes [16], the microridges arrangement either form a fingerprint-pattern over the keratinocytes surfaces or link to form a honeycomb appearance. The former author and others [55–57] using TEM reported that the microridges are composed of tiny villi-like projections resembling the intestinal epithelial microvilli.

Microridges have a number of potential functions, but their exact function is unknown [58]. The microridges may offer mechanical strength to prevent abrasion and structural support to steady the mucus epithelial layer [59]. The epidermal pattern provides a greater surface area that was hypothesized to play a role in mucus retention [60]. Similar tasks may be closed in more folded skin, where mucus and water may be held between the folds of the skin surface. The folded skin surface with abundant mucous cells of the fish may play a role in fish isolation and reflect a method of acclimatization to colder temperatures [23].

Intriguingly, the openings of mucous cells were more numerous in the adult fish than in the juvenile, more often in the dorsal skin than in the ventral skin in both age stages. There is evidence that the distribution of mucous cells is stress-sensitive [61, 62]. The number of the mucous cells increased in the skin after stress exposure, indicating a reaction in the barrier defense mechanism [23, 63–65]. On the other hand, Subramanian et al. [66] reported that the fish skin mucus plays a significant role in fish health since it is a crucial part of the innate immune system in fish and serves as the first physical and chemical barrier against infections. Moreover, fish skin is covered in mucous to adapt to the aquatic environment, which helps to preserve the skin's health, homeostasis, and integrity [11]. The differences in mucous cells number between the two studied stages may point to that the adult stage has greater receptivity to environmental stressors and the predilection of wild fish for a proactive fight response.

In earlier SEM reports, the sensory cells were more dominate in the juveniles than the adults [67]. This finding matched with our observation. The distribution of the sensory cells was more distributed in the juveniles by variable patterns on the dorsal and ventral skin. The sensory cells were dome-shaped with several pores. They are round-shaped with an elongated protrusion in farm fish [53]. While, in freshwater teleost, on SEM view, the superficial neuromasts are distinguished by many sensory hairs resembling long microvilli, that are covered by cupula [27]. These cells function and morphologically resemble those of the auditory and vestibular organs of vertebrates [68]. The sensory cells within the epidermal surface may act as chemo-mechanoreceptors.

Histological investigations of the current study showed few taste buds in the epidermis of the dorsal skin surface of the adult fish. Each taste bud consisted of basal cells, supporting cells, and outer sensory hairs. Similar findings were observed in *Mystus pelusius* [69]; Red-Tail Shark, *Epalzeorhynchos Bicolor* [70]; and also in *Eremophilus mutisii* fish [71].

As regards to the epidermal thickness, there is more information, and just one review found that the epidermal layer of the ventral skin is frequently thicker in benthic species [72] and in farmed fish gilthead seabream; *Sparus aurata* [53], similar to that mentioned in both studied age stages. In contrast, the obtained data from Mohamed et al. [73] indicated that the dorsal epidermal thickness is thicker than the ventral skin of two scaled fishes; Otolithes ruber and *Huso huso* and non-scaled fish; *Pangasius hypophthalmus*. In this respect, many authors [74–76] reported that the thickness of the skin epidermis is relied on the body part, age, sex, phases of the reproductive cycle, and seasonal changes and also depend on the diversity of epidermal cells of each part of the body [70, 77].

In the present study, both mucous and club cells were embedded in the epidermis of the juvenile and adult fish with different shapes and sizes. Numerous club cells were larger in their size and occupied the middle and superficial layer of the epidermis while the mucous cells were either rare or absent and also appeared obviously close to the outermost layer of the epidermis. These findings agree with the results reported in the red-tail shark by Mokhtar [70]. In common carp, it has been proposed that the high density of club cells serve as a defense mechanism to counterbalance the low density of the mucous cells [78]. Other studies supposed that in similar species, the club cells have a significant role in phagocytic functions [79, 80] or they can release alarm substance, which causes an alarm response [81–83], also they have a role as repair the injured mucosal and epithelial cells brought on by pathogenic agent [29].

Previously published data support our findings of the dermis, formed of two main layers: the outer stratum laxum layer (loose connective tissue with numerous melanophores below the basement membrane) and the inner stratum compactum layer (fibrous connective tissue with bony plates) [7, 53, 84–87].

Based on the dermis morphometrical analysis, the thickest dermal layer was recorded in the dorsal skin region of the adult fish while the thinnest was in the dorsal skin part of the juvenile fish. Dermal layer thickness is based on the collagen fibers which have a potential energy storage device giving fish skin an exotendon function [22, 88–90].

Melanocytes were observed at the dorsal skin below the basement membrane of both juvenile and adult fishes while few numbers were noticed at the ventral surfaces. Our results are in accordance with the reports of several authors [7, 53, 70, 91–93] who clarified the existence of the melanocytes in the dermal layer of the skin in some fish species. In vertebrates, the integument melanocytes exhibits various adaptive functions such as camouflage [94–96], defense [97–99], photo detection [96], mate selection [100, 101], and maintain body temperature [96].

Pit organs (neuromasts) were observed in the skin of the adult and juvenile fish. Similar results were found by Yang et al. [87] in Yangtze sturgeon (*Acipenser dabryanus*), and in Red-Tail Shark *Epalzeorhynchos Bicolor* [70]. Several studies explained the significant role of these organs as Engelmann et al. [102] who reported that the pit organs have an extremely sensitive function toward the velocity of water flow and vibration in stillwater. Others mentioned their mechanical, thermal and electrical pain functions [103]. Additionally, the pit organs exhibited sensory functions as they are able to convert mechanical stimulus into an electric signal [104, 105].

Histologically, several dermal bony plates with different shapes and sizes were demonstrated in the skin of both adult and juvenile fishes. The existence of the bony plates is associated and contact with many functions of protection, flexibility and toughness [106]. Several studies mentioned the existence of dermal bony plates in some fish species including *Acipenser dabryanus* [87], armored catfish *Pterygoplichthys pardalis* [107], *Ancistrus dolichopterus* [108], and Armored Siluriforms [109]. These bony plates may compensate for the functions of the scales in these species, as most of these fishes are scaleless.

## Conclusion

This research is the first to histologically and ultrastructurally characterize the dorso-ventral skin of the juvenile and adult starry puffer fish (*Arothron stellatus*, Anonymous 1798). Analysis of the epidermal thickness, microridges area of the epithelial cells clarified greater epidermal thickness in ventral skin in both of the studied stages. Larger and more defined microridges were seen in the dorsal skin than in the ventral skin of adult fish, contrary to juvenile ones. There was a variable distribution of the mucous, club, and sensory cells in the dorsal and ventral skin of juvenile and adult fish. The observed variations between the studied fishes may be a result of the many settings that the species inhabits and the various environmental difficulties that they confront.

In the future research, biochemical analysis of the juvenile and adult starry puffer fish skin should be carried out to clarify the degree of skin toxicity in each stage. The toxin (tetrodotoxin) may be also clarified its role in medical and therapeutic aspects. Additionally, starry puffer fish skin could represent a promising source for separating collagen and gelatin, to be utilized in the biomedical and pharmaceutical industries.

## Data Availability

Data are available from the corresponding author on reasonable request.
